# Gait analysis following single-shot hyaluronic acid supplementation: a pilot randomized double-blinded controlled trial

**DOI:** 10.1186/s40814-019-0443-4

**Published:** 2019-04-22

**Authors:** Luis Carlos Pereira, Claude Schweizer, Sara Moufarrij, Swenn M. Krähenbühl, Julien Favre, Gerald Gremion, Lee Ann Applegate, Brigitte M. Jolles

**Affiliations:** 1 Centre Hospitalier Universitaire Vaudois (CHUV), Site Hôpital Orthopédique, Avenue Pierre Decker 4, CH-1011 Lausanne, Switzerland; 2 Department of Musculoskeletal Medicine (DAL), University of Lausanne, Lausanne, Switzerland; 3 Swiss Biomotion Lab, Centre Hospitalier Universitaire Vaudois (CHUV), Site Hôpital Nestlé, Avenue Pierre Decker 5, CH-1011 Lausanne, Switzerland; 4 Centre for Applied Biotechnology and Nuclear Medicine, University of Zurich, Rämistrasse 71, CH-8006 Zurich, Switzerland

**Keywords:** Gait analysis, Hyaluronic acid, Injection, Knee, Osteoarthritis, Viscosupplementation

## Abstract

**Objectives:**

Viscosupplementation with new-generation, polyol-containing, cross-linked hyaluronic acid (HA) gels reduces joint inflammation in patients with knee osteoarthritis. Gait analysis is a complementary outcome measure to standard patient-reported scores and physical measures for testing the effect of HA injection. This three-arm, prospective, randomized, controlled, double-blind, feasibility pilot study investigated which gait parameters are more sensitive following a single bolus injection of polyol-containing HA for knee osteoarthritis.

**Methods:**

Twenty-two patients with Ahlbäck grade II–III knee osteoarthritis were randomly allocated into three groups: (1) HA + mannitol (*n* = 9), (2) HA + sorbitol (*n* = 5), and (3) saline placebo (*n* = 8). Patients were assessed by blinded observers prior to injection and at 4 weeks post-injection (4W). Outcome measures included the Western Ontario and McMaster Universities Osteoarthritis Index (WOMAC), Knee Society score (KSS), EuroQol in five-dimensions (EQ-5D), VAS pain, and VAS stiffness. Gait was assessed over 30 m using a portable inertial-based data logger (Physilog®).

**Results:**

Differences between 4W and baseline were statistically significant for the mannitol-containing viscosupplement, with a median increase of 0.076 m/s on gait speed (*p* = 0.039), 0.055 m on stride length (*p* = 0.027), and 15 points on the KSS (*p* = 0.047). In contrast, the HA + sorbitol and saline groups demonstrated no significant changes from baseline to 4W in any gait parameters or self-reported outcome measures (all *p* > 0.3). The observed increase in gait speed is approximately 13% greater than the mean difference between healthy subjects and those with knee osteoarthritis, is clinically important, and thus is a sensitive gait parameter.

**Conclusions:**

This study demonstrated gait speed and stride length are the most relevant gait parameters to investigate when assessing the effect of polyol-containing HA viscosupplementation. This study supports the need for a larger, randomized, controlled, clinical trial to assess the effect of a single-bolus HA injection versus multiple injections in people with knee osteoarthritis using both gait performance and self-reported parameters of knee function.

**Trial registration:**

This study was retrospectively registered at clinicaltrials.gov on August 20, 2018, and assigned #NCT03636971.

**Level of evidence:**

I

## Introduction

Knee osteoarthritis (OA) represents significant cost to society as a result of work absenteeism and treatment regimens [1, 2]. Options to alleviate pain and restore function include analgesics, anti-inflammatories, physiotherapy, viscosupplementation, and joint replacement for later stages of the disease [3]. Viscosupplementation consists of intra-articular injection of a hyaluronic acid (HA) compound that acts as a lubricant and shock absorber to increase resilience and promote joint health [4]. HA is a non-sulfated physiological linear glycosaminoglycan found in synovial fluid (3500 mg/kg) and cartilage surrounding chondrocyte cells (1200 mg/kg) [5]. HA maintains extracellular structure by holding moisture and maintaining viscoelasticity of tissue [4]. In cartilage, HA aggregates in the presence of aggrecan, hyaluronan and proteoglycan link protein 1 (HAPLN1), and water to provide adequate resistance to joint compression [6].

HA molecules in cartilage decrease in molecular weight with age, resulting in less support to the extracellular matrix [7]. Oxygen free radicals, found in diseased joints, depolymerize HA into oligosaccharides, which have a different viscosity and protective properties compared to normal synovial fluid [8]. Chemical modifications of viscosupplements with polyol free radical scavengers such as sorbitol and mannitol have been proposed to eliminate the oxygen free radicals found in diseased joints [9, 10]. These new generation non-crosslinked gels have a reduced risk of allergic reactions compared to HA derived from traditional sources such as rooster’s comb, chicken cartilage, or microbial fermentation have longer HA chains and a higher viscosity than first-generation in vitro-produced HA and lack a non-naturally occurring motif found in crosslinked HA that may trigger an immune reaction. The presence of a polyol stabilizes the HA and reduces joint inflammation. Consequently, new generation viscosupplements are expected to be more effective in diminishing the signs of joint inflammation [11].

Several systematic reviews have compared the effectiveness of viscosupplementation with placebo intervention [12]. However, these reviews evaluated trials using viscosupplements which have been commercially available for many years and did not include the newer generation of viscosupplements characterized by the presence of a polyol (i.e., sorbitol, mannitol) and higher concentrations of HA. Furthermore, new generation viscosupplements tend to be used in a “one-shot” injection clinical protocol. A single bolus has been demonstrated to trigger less inflammation and a reduced antibody response when compared to a series of three injections. [13] “One-shot” administration may offer other advantages in clinical settings as well, including a lower risk of infection, reduced cost, and improved convenience for the patient.

The effectiveness of viscosupplementation remains controversial. While the American Association of Orthopedic Surgeons (AAOS) and the National Institute for Health and Clinical Excellence (NICE) “cannot recommend using hyaluronic acid for patients with symptomatic OA of the knee” [14, 15], the European League Against Rheumatism (EULAR) advocates that “there is evidence to support the efficacy of HA in the management of knee OA, both for pain reduction and functional improvement” [16]. This debate is believed to be partially driven by the heterogeneity of the products currently being used and the incomplete understanding of how HA injections provide a therapeutic action [17]. A recent review described the mechanical and pharmacological stages of the proposed mechanism of exogenous HA activity in the knee [18].

The Osteoarthritis Society Research International (OARSI) recommends patient-reported and physical performance measures when conducting trials in OA [19]. Physical performance has traditionally been assessed by the timed up and go test, the 6-min walking test, and the stair climbing test [20]. Gait analysis has received less attention in trials studying the effects of viscosupplementation. Numerous studies have reported differences in gait biomechanics between people with knee OA and healthy controls [21]. Thus, analyzing gait may give rise to new perspectives when studying different therapeutic approaches. There is a paucity of trials that have studied the effects of one-shot polyol-containing viscosupplements on human gait.

The aim of this prospective, randomized, controlled, feasibility, clinical pilot study was to determine the most relevant gait parameters and clinical outcome measures when assessing the effectiveness of polyol-containing HA injection in people with knee OA.

### Patients and methods

This investigation was conducted in a university hospital setting. Patients with a diagnosis of primary OA of the knee, Ahlbäck grade II or III, and who provided informed consent were included. Exclusion criteria were a recent history of infection, diabetes, neurological impairment, chronic venous or lymphatic stasis, pain medication of level 2, 3, or 4 according to the World Health Organization analgesic ladder, [22] psychiatric conditions, contraindications for HA joint injection, and HA or steroid injections within the last 6 months.

At the initial screening appointment, all patients underwent an X-ray to determine the grade of OA. If all inclusion and exclusion criteria were respected, consecutive patients were invited to participate in the study. Following their approval, patients were contacted by telephone to schedule an appointment for the injection. On the first day, prior to the injection, patients underwent the baseline clinical assessment (i.e., outcome measure questionnaires and gait analysis), which was performed by a clinician blinded to the injection randomization. Patients were assigned to one of three groups according to a previously developed, computer generated, randomization key [23] and then received the injection. Each patient received one injection of either HA + mannitol (1800kD, Ostenil Plus®, TRB Chemedica SA, Switzerland) (group 1), or HA + sorbitol (2000 kD, Synolis®, Gebro-Pharma AG, Switzerland) (group 2), or the same volume of saline (group 3). Injections were conducted with a lateral approach by physicians who were advised of the product to be used immediately prior to the injection. The two physicians responsible for the injections did not participate in data collection or data analysis. Patients were reassessed by an independent observer at 4 weeks post-injection (4W), which is routine for all patients at our institution. The clinicians conducting the 4W follow-up were blinded to baseline scores and the product injected. Patient recruitment for this study began in May 2013, and the last follow-up appointment was completed in February 2015.

Outcome measures included the visual analog scale (VAS) question of the EuroQol in five dimensions questionnaire (EQ-5D-VAS) [24], VAS pain, VAS stiffness, the Western Ontario and McMaster Universities Osteoarthritis Index (WOMAC), [25], and the Knee Society score (KSS) [26]. The clinicians at our institution are familiar with these tools. Patients’ gait was assessed during two 30 m walks along an indoor corridor at each patient’s preferred speed. Patients were instructed to walk normally, and no verbal encouragement was given during the test. Miniature inertial sensors were mounted on the trunk (sacrum) and each thigh and shank to measure lower limb and trunk rotations, and these were linked to a portable data logger (Physilog, BioAGM, Switzerland). Temporal and spatial parameters were computed to determine four basic gait parameters: walking speed, stride length, cadence, and duration of the swing phase relative to the cycle duration, which were the primary outcomes for this study. These parameters were measured over the 30 m of the two trials according to a previously validated protocol [27]. Patients were asked directly about adverse events by the outcomes assessor at 1 day, 1 week, and 4W post-injection.

Research Ethics Board approval was obtained to conduct this randomized, controlled, clinical pilot study (CER-VD No 273/13).

### Data collection and analysis

The gait parameters measured in each cycle of the two walks were averaged during the steady part of the walks to provide a single measure of speed, stride length, cadence, and swing duration for each patient at baseline and at 4W. The changes between baseline and 4W were calculated individually for each patient, for the four gait parameters and five clinical outcome measure scores. Medians and interquartile ranges were used to report the changes from baseline to 4W within each patient group, as changes did not follow a normal distribution. Wilcoxon signed-rank tests were used to compare baseline and 4W measures.

**Fig. 1:**
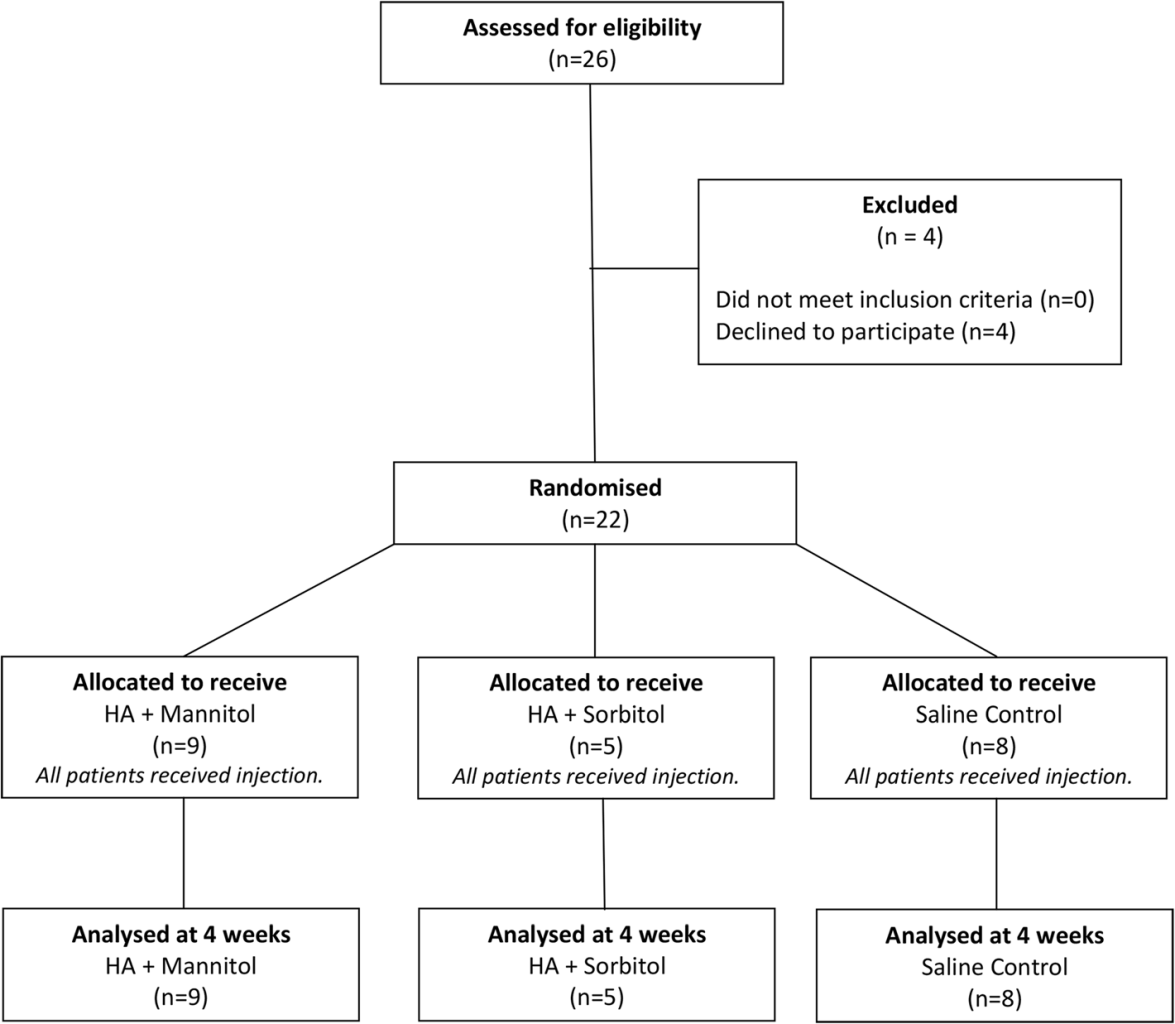
CONSORT patient dissemination flow chart

For this exploratory, prospective, randomized, double-blind, pilot study, a minimal sample size of twenty patients was selected to determine the feasibility of the trial design and select appropriate outcome measures for a future larger trial (Fig. 1). The limited sample size in this study did not allow for comparison of changes between patient groups.

All statistical analyses were performed with Matlab (Release 2014b, The MathWorks, USA) using a significance level set a priori to 5%.

## Results

Overall, 22 injections were performed: 9 in group 1 (HA + mannitol), 5 in group 2 (HA + sorbitol) and 8 in group 3 (saline control). Twelve patients were male and ten were female. Patients had a mean (standard deviation) age of 53.5 (11.89) years and BMI of 28.4 (4.1) kg/m^2^ (Table 1).

**Table 1:** Patient demographics

	HA + mannitol (*n* = 9)	HA + sorbitol (*n* = 5)	Saline (*n* = 8)	Total (*n* = 22)
Gender: male (*n*, %)	6 (67%)	1 (20%)	5 (63%)	12 (55%)
Gender: female (*n*, %)	3 (33%)	4 (80%)	3 (37%)	10 (45%)
Age (years), mean ± std. dev	50.8 ± 11.7	56.4 ± 13.0	54.8 ± 12.3	53.50 ± 11.9
Body mass index (kg/m^2^), mean ± std. dev	29.0 ± 4.6	26.8 ± 3.4	28.7 ± 4.0	28.4 ± 4.1

Gait parameters before and 4W after injection are summarized in Table 2.

**Table 2:** Gait parameters for patients who received injections of HA + mannitol, HA + sorbitol, or saline, pre-injection (visit 1) and 4 weeks post-injection (visit 2)

	HA + mannitol (*n* = 9)		HA + sorbitol (*n* = 5)		Saline (*n* = 8)	
	Baseline	4 weeks	Baseline	4 weeks	Baseline	4 weeks
Walking speed (m/s), mean (SD)	1.096 (0.211)	1.367 (0.183)	1.122 (0.213)	1.239 (0.175)	1.185 (0.219)	1.237 0.185)
Stride length (m), mean (SD)	1.314 (0.180)	1.367 (0.183)	1.217 (0.181)	1.239 (0.175)	1.273 (0.180)	1.311 (0.144)
Cadence (steps/minute), mean (SD)	49.764 (4.335)	50.986 (4.781)	55.011 (3.204)	54.175 (2.546)	55.579 (3.944)	56.426 (3.470)
Swing duration (% gait cycle), mean (SD)	40.835 (2.231)	39.673 (2.736)	41.000 (1.959)	40.735 (1.815)	39.936 (2.120)	40.245 (2.426)

**Fig. 2:**
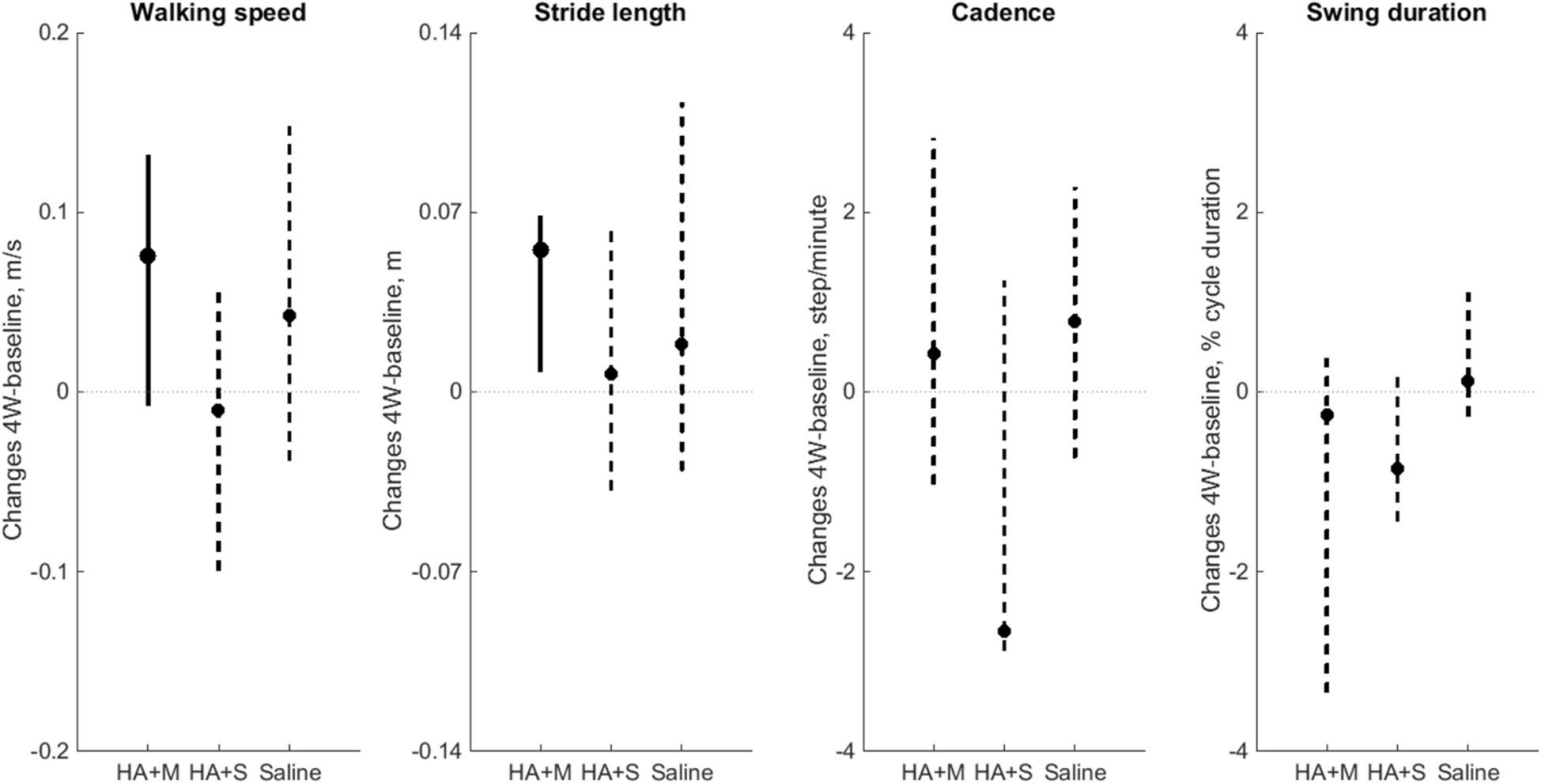
Changes in gait parameters from baseline to 4W post-injection (i.e., post-injection value minus baseline value) for the HA + mannitol group (HA + M), HA + sorbitol group (HA + S), and the saline group. Data are presented as medians (dot) and interquartile ranges (vertical lines). Significant changes between baseline and 4W are indicated with solid lines, whereas non-significant changes are indicated with dashed lines

Changes in walking speed and stride length from baseline to 4W were statistically significant for the HA + mannitol group, with median increases of 0.076 m/s (*p* = 0.039) and 0.055 m (*p* = 0.027), respectively (Fig. 2). Changes in cadence (*p* = 0.30) and in swing phase duration (*p* = 0.43) were not statistically different for the HA + mannitol group (Fig. 2). There were no statistically significant changes from baseline to 4W in any gait parameters in the HA + sorbitol and saline groups (*p* > 0.3).

**Fig. 3:**
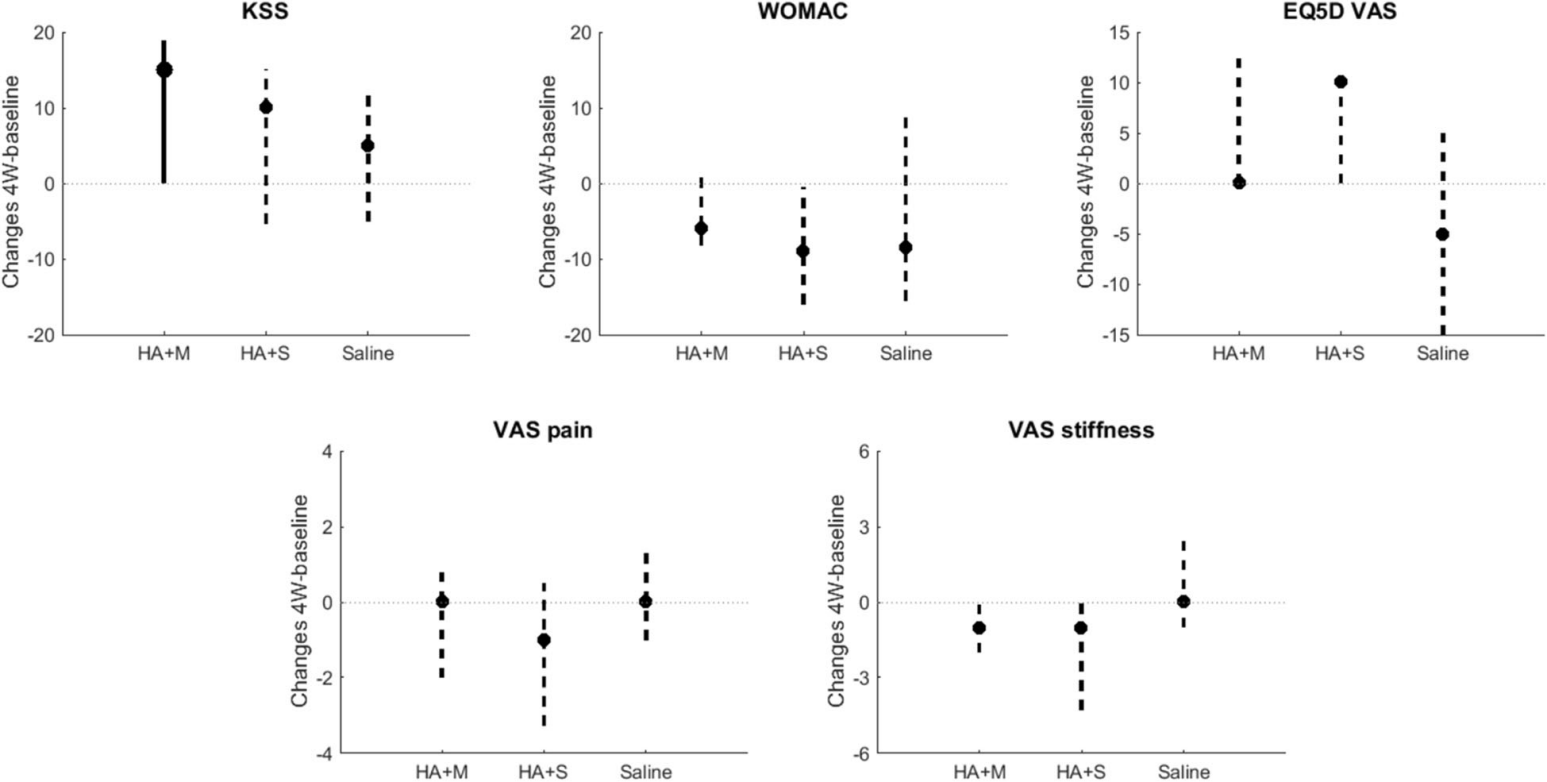
Changes in clinical outcome measure scores from baseline to 4W post-injection for the HA + mannitol group (HA + M), the HA + sorbitol group (HA + S), and the saline group. Data are presented as medians (dots) and interquartile ranges (vertical lines). Significant changes from baseline to 4W are indicated with solid lines, whereas non-significant changes are indicated with dashed lines

With respect to the clinical outcome measures, the change in KSS score from baseline to 4W was statistically significant for the HA + mannitol group, with a median increase of 15 points (*p* = 0.047) (Fig. 3). Changes in the other clinical scores for the HA + mannitol group and in all scores for the two other groups were not statistically significant (*p* > 0.1).

No adverse reactions were observed.

## Discussion

This prospective, randomized, controlled, exploratory pilot study found that gait speed and stride length are the most relevant gait parameters to investigate when assessing the effects of HA injection in people with knee OA.

A search of the literature produced seven trials that evaluated gait analysis following HA knee injection [28–34]. Two studies were excluded from further review: one focused on EMG muscular patterns; [33] the other [35] was a secondary report of a population already described elsewhere [29]. The five remaining trials were heterogeneous for the study design, outcomes chosen, methods of data collection, and substances delivered. Gait data were collected with a six-camera setup in two studies [29, 34], an eight-camera setup in one study [30], and a 10-m walkway system in another [31]. In the fifth study, Lester and Zhang [32] used a portable device with five inertial sensors, similar to the one used in the present trial. However, in that trial, gait was performed outdoors, and patients were asked to initially perform a 100-m warm-up walk before a second 100-m walk was performed to collect data. It is possible that walking 200 m may have induced fatigue in some patients, which may have negatively influenced the data analysis. The 60-m distance selected in the present study seemed to be a reasonable walking distance to assess patients with knee OA. Nevertheless, Lester and Zhang reported a walking speed of 1.12 m/s, a step length of 0.63 m, and a cadence of 106steps/min at 3 weeks after the final HA injection, which are equivalent to our findings. However, their trial did not have a control group for comparison. Of the other four studies, one did not report gait scores [30], one expressed the delta between follow-up moments for the gait speed (i.e., no absolute values) [31], one reported normalized gait velocity and step length according to body height [34], and Skwara et al. [29] presented absolute values for walking speed and stride length and observed a walking speed of 1.16 m/s, similar to that reported by Lester and Zhang. However, the results may not be comparable, since the follow-up assessment was performed at 12 weeks.

In the present study, the mean increase in gait speed (0.076 m/s) found in the HA + mannitol group at 4 weeks post-injection is thought to be clinically relevant. This increase in walking speed is 13% greater than the mean difference in walking speed between healthy subjects and those with knee OA, based on data from a recent meta-analysis [21]. Interestingly, increases in gait velocity and cadence have also been reported following a local injection of anesthesia in subjects with knee OA [36]. Thus, pain reduction is likely a key factor in the efficacy of viscosupplementation in improving gait.

Four of the previous studies used multiple injections of HA. Only the present trial and the study by Skwara et al. [29] evaluated the effects of a single bolus of viscosupplementation through gait analysis. Repeated viscosupplementation not only improves knee OA symptoms between injection cycles, but also exerts a marked carry-over effect for at least 1 year post-administration [37]. Delivering a single injection allows for shorter follow-up periods and potentially decreased overall healthcare costs. Based on clinical experience, we believed that a single bolus injection could significantly change gait pattern and alleviate patients’ symptoms. The results of this pilot study support this hypothesis and the need for a large, randomized, controlled, clinical trial to assess the effectiveness of a single-bolus HA injection in people with knee OA. This hypothesis is also supported by a pilot, open, non-comparative study that demonstrated reduced joint pain and increased function in 79 patients with knee OA at 6 months following a single intra-articular injection of non-crosslinked HA [38]. HA injections studied a decade ago did not employ the same compounds used today. Viscosupplements are continually evolving; the newer products with stabilizer additives are expected to exert an anti-free radical effect, which could potentially decrease a local inflammatory reaction in the knee and impart greater efficacy with a single intra-articular injection.

While gait patterns demonstrated statistically significant differences at 4 weeks following HA injection compared to baseline, there were no significant differences in self-reported WOMAC, EQ-5D-VAS, VAS pain, or VAS stiffness scores in the present trial. A 4-week assessment was deemed adequate, as the intra-articular half-life of HA is less than 2 days. In contrast, Kotevoglu et al. [35] reported improvements in WOMAC physical function and WOMAC stiffness scores induced by both sodium hyaluronate and hylan G-F 20 at 1 month post-injection in a randomized clinical trial of 59 patients that remained significant at 6 months (*p* < 0.01). Thus, a single HA injection appears to have a positive impact on pain and function up to 6 months following injection. This is corroborated by electromyography, in which HA injection effectively decreased co-contraction and improved motor activity of the lower extremity muscles for up to 6 months [33]. The absence of consistent significant differences in self-reported outcome measures and the presence of statistically significant differences in gait patterns during walking tests underline the importance of using gait analysis when assessing the management of knee OA. In a gender-specific study of OA of the knee, patient-reported measures only partially reflected sex differences consistently observed with physical impairment and performance-based measures [20]. Thus, a comprehensive functional assessment in patients with OA should include physical impairment, performance, and self-reported parameters of knee function.

This pilot study was designed to evaluate the feasibility of scientific outcomes, i.e., the gait parameters most relevant for the assessment of polyol-containing HA injection in people with knee OA. According to Thabane et al. [39], pilot studies can also assess feasibility outcomes for the study process, resources, and management. A secondary feasibility outcome around this study’s process, specifically patient recruitment, became evident during its conduct. Many patients were unwilling to participate, as they did not want to risk being allocated to the placebo group because they were in pain, a common concern in trials investigating OA interventions [40]. In contrast to other trials, [31] the present study did not introduce any rescue medication following the injection, to avoid additional confounding variables. The pain management protocol will be reviewed and may be revised for future trials, to perhaps compare outcomes of a single HA injection versus multiple injections and avoid having a placebo group.

This study has limitations. A sample size of 22 patients is informative for evaluating feasibility outcomes, but is a limitation for statistical analysis, even in a pilot study. Based on the results presented herein, power calculations indicate that a future trial designed to detect a difference between groups of 0.076 m/s on walking speed, 0.055 m on stride length, and 15 points on the KSS would require a sample of 37, 26, and 17 participants per group, respectively (alpha 0.05, beta 0.8). The HA + sorbitol group included only 5 participants, compared to 9 and 8 participants in the other two groups, limiting comparisons. Also, the HA + mannitol group was, on average, 4 to 5 years younger than the other two groups, which may have contributed to the observed improvements in gait speed and stride length. The present study did not encourage the use of a medication diary during the follow-up period. Including an observation period of 1–2 weeks may have helped to distinguish the “trial” effect from placebo, or the attention control effect. Intrinsic patient factors such as obesity, anxiety, and depression are also associated with poor WOMAC scores and walking times [41, 42]. This study ensured that BMI was similar among all three groups, but it did not account for psychological factors such as anxiety and depression that could influence the final outcome.

## Conclusions

Gait analysis is proposed as a complementary outcome measure to the standard patient-reported scores and physical measures for testing the effectiveness of HA injection, with gait speed and stride length as the most relevant parameters for investigation. The results of this study support the need for a larger, randomized, controlled, clinical trial, with at least 37 participants per group, to assess the effectiveness of a single-bolus HA injection versus multiple injections in people with knee OA using both gait performance and self-reported parameters of knee function.
